# Comparison of Bioelectrical Impedance Analysis, Slaughter Skinfold-Thickness Equations, and Dual-Energy X-ray Absorptiometry for Estimating Body Fat Percentage in Colombian Children and Adolescents with Excess of Adiposity

**DOI:** 10.3390/nu10081086

**Published:** 2018-08-14

**Authors:** Katherine González-Ruíz, María Medrano, Jorge Enrique Correa-Bautista, Antonio García-Hermoso, Daniel Humberto Prieto-Benavides, Alejandra Tordecilla-Sanders, César Agostinis-Sobrinho, María Correa-Rodríguez, Jacqueline Schmidt Rio-Valle, Emilio González-Jiménez, Robinson Ramírez-Vélez

**Affiliations:** 1 Centro de Estudios Para la Medición de la Actividad Física CEMA, Escuela de Medicina y Ciencias de la Salud, Universidad del Rosario, Bogotá 111221, Colombia; katherine.gonzalez@docentes.umb.edu.co (K.G.-R.); jorge.correa@urosario.edu.co (J.E.C.-B.); danielprietob@gmail.com (D.H.P.-B.); alesanders_0615@hotmail.com (A.T.-S.); 2 Grupo de Ejercicio Físico y Deportes, Facultad de Salud, Programa de Fisioterapia, Universidad Manuela Beltrán, Bogotá 110231, Colombia; 3 Institute for Innovation & Sustainable Development in Food Chain (IS-FOOD), Public University of Navarra, Campus de Arrosadía, 31006 Pamplona, Spain; maria.medrano.echeverria@gmail.com; 4 Laboratorio de Ciencias de la Actividad Física, el Deporte y la Salud, Facultad de Ciencias Médicas, Universidad de Santiago de Chile, USACH, Santiago 7500618, Chile; antonio.garcia.h@usach.cl; 5 Faculty of Health Sciences, Klaipeda University, Klaipeda 92294, Lithuania; cesaragostinis@hotmail.com; 6 Departamento de Enfermería, Facultad de Ciencias de la Salud, Avda. De la Ilustración, 60, University of Granada, Granada 18071, Spain; macoro@ugr.es (M.C.-R.); jschmidt@ugr.es (J.S.R.-V.); emigoji@ugr.es (E.G.-J.)

**Keywords:** adiposity, body composition, validation study, DXA, children, adolescents

## Abstract

Dual-energy X-ray absorptiometry (DXA) has been considered a reference method for measuring body fat percentage (BF%) in children and adolescents with an excess of adiposity. However, given that the DXA technique is impractical for routine field use, there is a need to investigate other methods that can accurately determine BF%. We studied the accuracy of bioelectrical impedance analysis (BIA) technology, including foot-to-foot and hand-to-foot impedance, and Slaughter skinfold-thickness equations in the measurement of BF%, compared with DXA, in a population of Latin American children and adolescents with an excess of adiposity. A total of 127 children and adolescents (11–17 years of age; 70% girls) from the HEPAFIT (Exercise Training and Hepatic Metabolism in Overweight/Obese Adolescent) study were included in the present work. BF% was measured on the same day using two BIA analysers (Seca^®^ 206, Allers Hamburg, Germany and Model Tanita^®^ BC-418^®^, TANITA Corporation, Sportlife Tokyo, Japan), skinfold measurements (Slaughter equation), and DXA (Hologic Horizon DXA System^®^, Quirugil, Bogotá, Columbia). Agreement between measurements was analysed using *t*-tests, Bland–Altman plots, and Lin’s concordance correlation coefficient (*ρ*c). There was a significant correlation between DXA and the other BF% measurement methods (*r* > 0.430). According to paired *t*-tests, in both sexes, BF% assessed by BIA analysers or Slaughter equations differ from BF% assessed by DXA (*p* < 0.001). The lower and upper limits of the differences compared with DXA were 6.3–22.9, 2.2–2.8, and −3.2–21.3 (95% CI) in boys and 2.3–14.8, 2.4–20.1, and 3.9–18.3 (95% CI) in girls for Seca^®^ mBCA, Tanita^®^ BC 420MA, and Slaughter equations, respectively. Concordance was poor between DXA and the other methods of measuring BF% (*ρ*c < 0.5). BIA analysers and Slaughter equations underestimated BF% measurements compared to DXA, so they are not interchangeable methods for assessing BF% in Latin American children and adolescents with excess of adiposity.

## 1. Introduction

Overweight and obesity in children and adolescents have emerged as serious public health concerns in both developed and developing countries [1]. An estimated 10% of schoolchildren worldwide are overweight, and a quarter of this group are obese [2]. Notably, a study published in 2014 and conducted in Latin American concluded that one in five children and adolescents were overweight or obese [3]. The increasing prevalence of childhood obesity, a metabolic disorder characterized by the accumulation of excess body fat, has been associated with several metabolic and cardiovascular diseases, such as type 2 diabetes mellitus, non-alcoholic fatty liver disease, and dyslipidemia [4,5]. Thus, body fat percentage (BF%) has been proposed as a risk factor for metabolic and cardiovascular disorders [6] and, therefore, there is a growing interest in the accurate measurement of BF%, rather than the conventional body mass index (BMI), as it might provide clinically useful insight.

There is currently a wide variety of methods available for determining BF%, including relatively simple field techniques, such as bioelectrical impedance analysis (BIA) and skinfold-thickness measurements, as well as more sophisticated instruments including dual-energy X-ray absorptiometry (DXA) [7]. DXA has been considered a reference method for measuring BF% in children and adolescents with excess of adiposity [8] thanks to the long-term precision of the values it yields (the coefficient of variation, CV, of repeated measurements is 2%) [9]. However, its use is limited in a clinical setting because the machine is bulky, immobile, expensive, requires technical expertise, and involves exposure to ionizing radiation. Considering that DXA is impractical for routine field use, it would be convenient to develop other methods of accurately measuring BF% in children and adolescents with excess of adiposity.

BIA analysers, including foot-to-foot and hand-to-foot methods, have widely been used to make non-invasive BF% measurements in children [10]. The advantages of BIA technology include its portability, simplicity, speed, safety, and low cost, and since their use requires minimal subject collaboration they are ideal for routine practice in children and adolescents [11]. The BIA method has proven to provide acceptable quantification of BF% in children from the general population [10,12], but the validity of BIA analysers is influenced by sex, age, ethnicity, and level of body fat [13,14]. It has been suggested that the validity of BIA might be limited in obese people because they have an altered body geometry and body water distribution [15], however, there is no conclusive evidence of this theory. Given the convenience offered by BIA analysers, they have become increasingly popular among health professionals and their increasing availability might make them a suitable tool for screening obesity in children and adolescents. However, the accuracy of this technique has not been widely studied in children and adolescents with overweight/obesity or excess of adiposity [16,17,18].

On the other hand, predictive equations such as the Slaughter equations, which are based on sex, maturity, and skinfold thicknesses [19], have also been developed as an alternative for estimating body fat percentages in children [20,21]. Predictive equations might be useful as convenient screening tools to assess BF% at the primary health care level. However, they are generally population-specific and may only prove useful in populations with characteristics similar to those of the reference populations. Therefore, the development of predictive equations cannot be generalized to diverse populations.

To date, only a few studies, which reported mixed findings, have validated BIA analysers and the skinfold-thickness equations as methods for measuring BF% in different populations of children and adolescents with excess of adiposity compared to a reference method, such as DXA [22,23,24,25]. Variations in fat distribution patterns observed among ethnic groups imply that ethnicity plays an important role and should be considered when validating BF% [26,27,28]. However, to the best of our knowledge, there are no previous studies conducted in Latin American youths, as most authors have concentrated on Western and Asian populations. 

Since the prevalence of childhood obesity is increasing rapidly worldwide, accurate BF% measurement is of special interest for clinical practice and research purposes. On this basis, we aimed to compare the accuracy of BIA analysers, including foot-to-foot and hand-to-foot impedance, and Slaughter skinfold-thickness equations against DXA techniques for measuring BF% in a population of Latin American children and adolescents with excess of adiposity.

## 2. Methods

### 2.1. Study Design, Setting, and Participants

A baseline analysis of the clinical trial Exercise Training and Hepatic Metabolism in Overweight/Obese Adolescent (HEPAFIT) ClinicalTrials.gov Identifier: NCT02753231 was carried out between October 2017 and January 2018. The analysis involved a total of 127 children and adolescents (70% girls) aged 11–17 years who had an axiological evaluation of cardiovascular risk factors by primary care paediatric screening. The study received ethical approval from the Medical Research Ethics Committee of The University of Rosario (no. UR-21042016). All participants were informed of the study’s goals, and written informed consent was obtained from participants and their parents or legal guardians.

Details of background and design methods (i.e., characteristics of participants, sample calculation, randomization, outcomes and analysis plan), of the HEPAFIT Study had been previously published elsewhere [29], nevertheless, the most relevant information is briefly described below.

The following inclusion criteria were adopted: primary overweight/obese status, defined according to the International Obesity Task Force (IOTF) [30], or excess of adiposity (body fat > 30% by DXA), inactivity (no participation in exercise more than once a week in the previous six months), and having at least one parent or caregiver willing to participate in the program sessions. Those youths identified as being willing and almost immediately available were enrolled. Eligible adolescents for the study and those interested in participating were invited to a pre-test, which included a medical interview at the Centre of Studies in Physical Activity Measurements (in Spanish, CEMA), School of Medicine and Health Sciences, University of Rosario, Bogotá, Colombia.

### 2.2. Anthropometric and Body Composition Measures Parameters

Variables were collected at the same time, between 7:00 and 9:00 a.m., following an overnight fast. Body mass (kg) was measured using an electric scale (Model Tanita^®^ BC-418^®^, TANITA Corporation, Sportlife Tokyo, Japan; technical error of measurement (TEM = 0.719) with a range of 0–200 kg and accurate to within 100 g). Individuals wore only their underwear. Height and sitting height were measured with a portable stadiometer with a precision of 0.1 mm and a range of 0–2.50 m (Seca^®^ 206, Allers Hamburg, Germany; TEM = 0.001/0.115). Body mass index (BMI) was calculated as weight (kg)/height (m^2^), and BMI-z score was calculated using WHO Anthro-Plus 1.0.4 software^®^ (World Health Organization, Geneva, Sweden). Then, subjects were classified as children with overweight or obesity according to the IOTF BMI category [30]. The mean of two measurements of subscapular, triceps, and calf skinfolds was calculated in all participants using Holtain^®^ skinfold calipers (Holtain Ltd., Crymych, UK) in accordance with the methods of auxological anthropometry [31]. Anthropometric variables were measured by a Level 2 expert certified by the International Society for the Advancement of Kinanthropometry (ISAK) in accordance with ISAK guidelines [32]. The same trained investigator made all anthropometrics measurements. 

A tetrapolar whole body impedance meter foot-to-foot (Model Tanita^®^ BC 420MA) and a hand-to-foot (Model Seca^®^ mBCA 514 Medical Body Composition Analyzer) were used to analyse body fat percentage with the manufacturer’s equations, similar to previous studies [18]. Hand-to-foot considers the resistance of both the upper and lower body and trunk, whereas foot-to-foot only consider leg and the lower part of the trunk resistance [33]. The single-frequency foot-to-foot BIA devices provides estimated values for BF% calculated by passing an alternating current through the subject to measure the water content. The body fat is obtained by subtracting to the weight of fat-free mass to the total body water. For the Tanita^®^ BC 420MA measurements, the subject stood in an upright position with bare feet on the analyser footpads. The impedance between the two feet was measured while an alternating current (50 kHz and ∼ 200 μA) passed through the lower body. 

The Seca^®^ mBCA 514 is an eight electrode segmental multi-frequency analyser that measures impedance at 19 frequencies ranging from 1 kHz to 1 MHz. It is a ‘stand-on’ multi-frequency BIA device where subjects place their feet on top of the electrodes so that the heel is central to the smaller posterior electrode and the forefoot is central to the larger anterior electrode [34]. Each side of the handrail has six electrodes, two are chosen dependent on the height of the subject with the angle between arms and the body about 30°. The hands touch the electrodes so that the electrode separator is positioned between the middle and ring finger. Likewise, BF% was calculated by passing an alternating current through the subject to measure the water content. BIA values obtained at 5 and 50  kHz are used in the predictive equations. The root mean square error (RMSE) for this device has been reported to be 1.34  kg (total body water) and 0.79  kg (extracellular water) [35]. To ensure data quality, the equipment was calibrated daily using a known calibration standard. 

The reproducibility of the variables estimated by Seca^®^ mBCA 514 and Tanita^®^ BC 420MA were determined by the coefficient variation (CV%) and the TEM, based on the test-retest realized with 50 subjects out of the population of this study. The CV% were 5.5% (Tanita^®^ BC 420MA) and 5.9% (Seca^®^ mBCA 514), and the TEM were 0.001 kg (Tanita^®^ BC 420MA) and 0.002 kg (Seca^®^ mBCA 514), respectively. These BIA models have been previously reported to be valid and reliable methods of estimating body composition in population of children and adolescents [36,37].

Whole-body DXA was performed using a Hologic machine (Hologic Horizon DXA System^®^, Quirugil, Bogotá, Columbia) with Discovery software, version 12.3 (Bellingham, WA, USA). Scans were performed by the same trained operator, according to the laboratory standard protocol. The subjects were instructed to assume a supine position with their arms by their sides and palms in a neutral position. To ensure data quality, the equipment was calibrated daily using a known calibration standard following manufacturer instructions (step phantom scan for body composition calibration). The reproducibility of the variables estimated by DXA was determined by the CV% and the TEM, based on the test-retest realized with 20 subjects out of the population of this study. The CV% were 1.5%, and 0.011 g respectively. DXA has reported to provide acceptable accuracy and reliability in measuring body composition among children [38,39]. BF% by DXA was calculated as (fat mass/(fat mass + bone-free lean tissue mass + bone mineral content) × 100). Exchange of each site’s calibration spine phantom confirmed the reliability of pooling results from the three scanners.

### 2.3. Body Fat Calculation

The original Slaughter equations [19] based on the triceps and subscapular skinfolds were used to estimate BF% for each child. Slaughter equations have been reported to be a valid method for estimating body composition in children and adolescents [40,41]. Triceps and subscapular skinfold thickness were measured by highly trained and standardized technicians following recommended protocols [42]. Skinfold thicknesses were measured at the left side of the body to the nearest 0.1 mm using a Holtain skinfold calliper (Holtain Ltd., Crymych, UK) at the following sites: (1) triceps, halfway between the acromion process and the olecranon process, (2) subscapular, approximately 20 mm below the tip of the scapula, at an angle of 45° to the lateral side of the body, and (3) calf, at the level of maximum calf circumference, on the medial aspect of the calf. TEM was 0.431% for the triceps skinfold, 2.779% for the subscapular skinfold and 1.872% for the calf skinfold. Reliability was performed in a sample of 229 participants (median values for age = 12.8 ± 2.5 years, weight = 45.3 ± 11.1 kg, height = 1.49 ± 0.1 m, and BMI = 20.1 ± 3.3 kg/m^2^). Each observer measured each child three consecutive times within 1 h for the intra-observer assessment, while an inter-observer reliability investigation was performed on a separate day. The corresponding intra-observer technical error (reliability) of the measurements was 0.97% for the triceps skinfold, 0.98% for the subscapular skinfold [43], and 0.99% for the calf skinfold. Note that skinfold measurements have been reported to provide valid and reliable BF% estimation in paediatric population [21].

Somatic maturity was estimated from the equations proposed by Mirwald et al. [44]. In these equations, years from peak height velocity (PHV) were predicted by using chronological age and height, sitting height, and leg length. 

Pubertal stage (breast and pubic hair development for girls, genital and pubic hair development for boys; ranging from stage I to V) was self-assessed by the participants according to the classification according to the criteria of Tanner and Whitehouse [45].

### 2.4. Statistical Analysis

Chi-square and independent Student´s *t*-tests were used to calculate differences in categorical (genital and pubic pubertal stage) or continuous (remainder of the variables) variables between boys and girls, respectively. The average and standard deviation (SD) of the measurement for each method was calculated and differences between measurements were analysed using *t*-test for dependent samples. Reliability between DXA, BIAs (Model Seca^®^ mBCA 514 Medical Body Composition Analyzer and Model Tanita^®^ BC 420MA) and the Slaughter equation (skinfolds) estimates for BF%, was assessed by comparing and correlating repeat measurements, using the *t*-test for dependent samples and Pearson correlation coefficients, respectively. Bland and Altman [46] figures were created, in which differences between DXA vs BIA´s (Model Tanita^®^ BC 420MA and Model Seca^®^ mBCA 514 Medical Body Composition Analyzer) and DXA vs. Slaughter estimates, were plotted. Limits of agreement were calculated as mean difference ± 1.96 SD. The Lin’s concordance correlation coefficient (*ρ*c) was computed [47]. A value of 1 indicates perfect agreement; *ρc* values of <0.90 were considered to indicate poor agreement, *ρc* values of between 0.90 and 0.95 moderate agreement, *ρc* values of between 0.95 and 0.99 substantial agreement, and values >0.99 excellent agreement. Additionally, we have conducted Bland-Altman analysis to examine the associations between the difference of methods and the mean of each method (DXA vs. Seca^®^ mBCA 514; DXA vs. Tanita^®^ BC 420MA and DXA vs. Slaughter equations). Analyses were performed using the Statistical Package for Social Sciences (SPSS, version 20.0 for Windows; IBM Corporation, New York, NY, USA), and the level of significance was set at α = 0.05. The MedCalc Software version 17.2 (MedCalc Software BVBA, Ostend, Belgium) was used to assess the *p*c.

## 3. Results

Descriptive characteristics of the study participants stratified by sex are presented in Table 1. The mean BMI was 24.2 (2.5) kg/m^2^ in boys and 23.5 (4.1) kg/m^2^ in girls. As expected, significant differences were observed between boys and girls with respect to waist circumference (*p* = 0.009) and z-BMI (*p* = 0.013). Boys were found to have significantly lower values of BF% as measured by DXA, Seca^®^ mBCA 514, and Tanita^®^ BC 420MA compared to girls (*p* < 0.001 for all). In contrast, no significant differences were observed between genders for BF% estimated by Slaughter skinfold-thickness equations.

Univariate correlations revealed a significant association between DXA and the other methods used to measure BF% for both sex. In boys, BF% assessed by DXA correlated significantly with BF% assessed by Seca^®^ mBCA 514 (*r* = 0.726, *p* < 0.001), Tanita^®^ BC 420MA (*r* = 0.430, *p* = 0.005), and Slaughter equations (*r* = 0.532, *p* < 0.001). Similarly, in girls, BF% assessed by DXA correlated significantly with BF% determined by Seca^®^ mBCA 514 (*r* = 0.846, *p* < 0.001), Tanita^®^ BC 420MA (*r* = 0.652, *p* = 0.005), and measuring skinfolds (*r* = 0.711, *p* < 0.001).

Table 2 shows the mean BF% difference and *ρ*c between DXA and Seca^®^ mBCA 514, Tanita^®^ BC 420MA, and skinfold-thickness equations by sex. There were significant differences between BF% measured by DXA and the rest of the methods (Seca^®^ mBCA 514, Tanita^®^ BC 420MA, or Slaughter equations) in both boys and girls (*p* < 0.001), indicating a low agreement with DXA. Additionally, *ρ*c was poor between DXA and the other methods used to determine BF% in both boys and girls.

**Figure 1:**
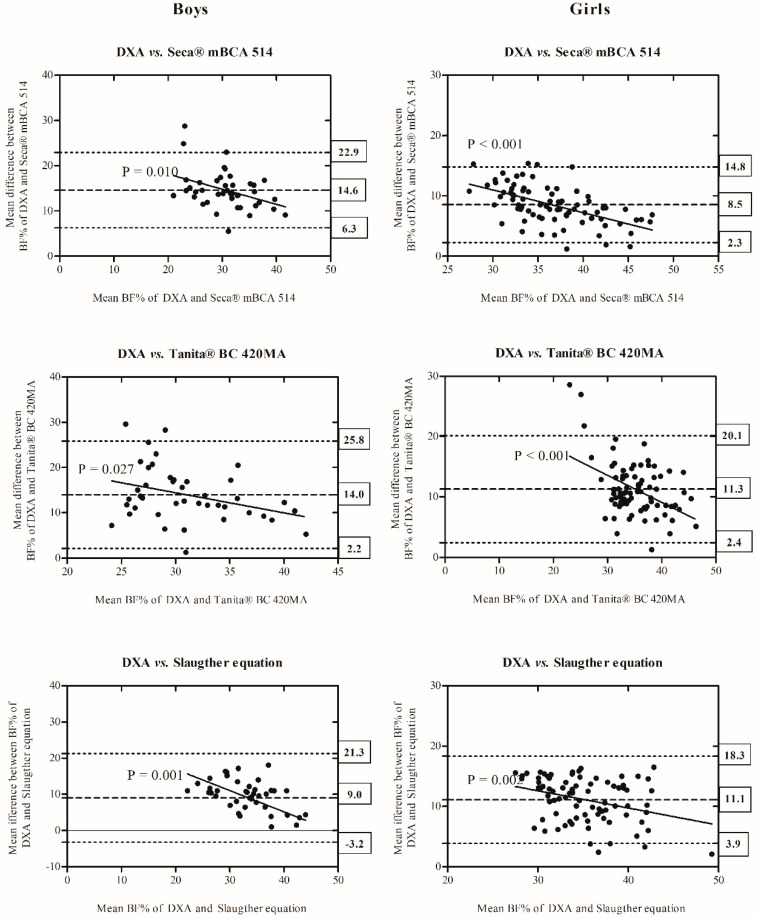
Comparison of BF% measured by DXA in comparison with Seca^®^ mBCA 514, Tanita^®^ BC 420MA, and Slaughter equations and displayed as Bland–Altman plots in boys and girls. The central line represents the mean bias between measurements. Dotted lines represent upper and lower limits of agreement. Y-axis represents difference of BF% measured by DXA minus BF% measured by Seca^®^ mBCA 514, Tanita^®^ BC 420MA or from skinfolds, respectively. The solid line and equation in each plot represent the linear regression between biases and body fatness by each method (DXA vs. Seca^®^ mBCA 514; DXA vs. Tanita^®^ BC 420MA and DXA vs. Slaughter equations). Abbreviations: BF%: body fat percentage; DXA: dual energy X-ray absorptiometry.

Comparisons of BF% derived from DXA compared to Seca^®^ mBCA 514, Tanita^®^ BC 420MA, or Slaughter equations are depicted in the Bland–Altman plots for boys and girls shown in Figure 1. In boys, the bias was 14.6 (6.3–22.9, 95% CI) in Seca^®^ mBCA 514, 14.0 (2.2–25.8, 95% CI) in Tanita^®^ BC 420MA, and 9.0 (3.2–21.3, 95% CI) in Slaughter equations, in relation to DXA. In girls, Bland–Altman plots show a bias of 8.5 (2.3–14.8, 95% CI), 11.3 (2.4–20.1, 95% CI), and 11.1 (3.9–18.3, 95% CI) in Seca^®^ mBCA 514, Tanita^®^ BC 420MA, or Slaughter equations, respectively, indicating limited agreement between DXA and the other methods of measuring BF%. The trend between the difference of methods and the mean of each method was statistically significant (DXA vs. Seca^®^ mBCA 514, *R*^2^ = −0.156 to −0.312; DXA vs. Tanita^®^ BC 420MA, *R*^2^ = −0.117 to −0.206, and DXA vs. Slaughter equations, *R*^2^ = −0.110 to −0.245, all *p* < 0.03), indicating a greater underestimation of BF% as body fat decreased.

In both paired *t*-tests (Table 2) and Bland–Altman plots (Figure 1), the results show that Seca^®^ mBCA 514, Tanita^®^ BC 420MA, and Slaughter skinfold-thickness equations significantly underestimated BF% in both sexes with respect to DXA.

## 4. Discussion

The main findings of this study show that BIA analysers and Slaughter skinfold-thickness equations provide less accurate measurements of BF% compared to DXA in children and adolescents with excess of adiposity. Therefore, Seca^®^ mBCA 514, Tanita^®^ BC 420MA, and Slaughter equations and not interchangeable with DXA for determining BF%. Despite the good repeatability of measurements, the Seca^®^ mBCA 514, Tanita^®^ BC 420MA, and Slaughter skinfold-thickness equations methods tested in this study cannot be recommended for measuring BF% in Latin American children and adolescents with excess of adiposity.

This study showed that Seca^®^ mBCA 514 and Tanita^®^ BC 420MA underestimated BF% in comparison with DXA. The Bland–Altman plots indicated poor agreement between both BIA analyzers and the reference DXA technique. Thus, neither foot-to-foot BIA (Tanita^®^ BC 420MA) nor hand-to-foot BIA (Seca^®^ mBCA 514) are suitable replacements for DXA in the assessment of BF% in children and adolescents with excess of adiposity from Latin America. Similarly, it has been previously reported that BIA is less accurate when measuring obesity [16,24,36,48]. In agreement with our results, Wan et al. [49] recently reported that BIA could be a valuable clinical tool to measure body composition at the group level, but is inaccurate for the individual obese adolescent. Eisenkölbl et al. [24] also found that a BIA analyser (2000M, Data Input GMbH, Germany) underestimated BF% compared to DXA in a sample of children and adolescents (6–18 years). Similarly, in three separate studies conducted in adolescents with excess of adiposity and weight (10–17 years across the three studies), Lazzer et al. [23,25,50] found that different methods of BIA (hand-to-foot or foot-to-foot Tanita [50]; different frequency BIA analysers [25]) underestimated BF% compared to DXA (Hologic QDR-4500 [25,50] or GE/Lunar Prodigy densitometer [23]), suggesting that these methods are not interchangeable. Differences in BF% as measured by BIA and DXA could be due to overestimation of fat-free mass hydration in obese subjects, which might result in underestimations of the BF% [15,51]. It has been assumed that total body water of lean body mass is 73.2% [52]. However, the total body water could be higher in children [53] and obese subjects [24,51], which could cause an overestimation of the lean body mass and, consequently, an underestimation of BF% [51] measured by BIA. Similarly, extracellular water is higher in obese adults [54] and children [51,55], which could also lead to an underestimated value for the BF%. Moreover, differences reported between DXA and BIA methods to estimate BF% might be due to the fact that BIA devices rely on the two-component (2-C) model, whereas DXA uses a three-component (3-C) model. Note that the 2-C model is directly affected by water and electrolytes, whereas the 3-C model is not [56,57]. In contrast, a study conducted in white and African American children and adolescents concluded that Tanita SC-240 has an acceptable accuracy for estimating BF% compared to DXA [36]. Considering the important role of ethnicity in body fat distribution [28], these contradictory results could be explained by differences across ethnic populations.

The mean differences in BF% obtained with the Seca^®^ mBCA 514 and Tanita^®^ BC 420MA analyzers compared to DXA techniques were much greater in boys than in girls. This could be due to sex variations in the prevalence of different types of obesity (total and central obesity) since it has been established that women have more generalized obesity, but less central obesity, than men [58]. These findings are similar to those obtained in previous studies investigating the reliability of BIA methods in adolescents with excess of weight [17,24,25,36,50].

On the other hand, we observed significant differences in BF% calculated by DXA and Slaughter skinfold-thickness equations, indicating that Slaughter equations would be poorly predictive of BF% in youths with excess of adiposity. Lazzer et al. [25] found high agreement limits between BF measured by DXA (Hologic Inc., Bedford, MA, USA) and Slaughter equations in Bland–Altman plots. Nevertheless, like our results, they found that Slaughter equations underestimated BF% (by 2.1 ± 5.0 kg in boys and 2.3 ± 3.5 kg in girls) in a sample of 43 overweight or obese adolescents (aged 14.2 ± 1.4 years) [25]. In a study by Freedman et al. [59], performed in non-obese and obese children, they also found significant differences in children with obesity when comparing BF% assessed by DXA (Norland XR36 pencil-beam, CT) or Slaughter equations (Bland–Altman plot). However, they found that Slaughter equations overestimated BF% measured by DXA in children with a high body fat [59]. The differences in our study between BF% measurements obtained by these two methods could be because the Slaughter equations were developed in a sample with low levels of body fat (for example, in the sample used by Slaughter et al. [19] to develop their equations the value of the sum of triceps and subscapular folds of postpubescent girls was 26.8 ± 9.2 mm, whereas the sum of triceps and subscapular folds of postpubescent girls in our sample was 37.2 ± 8.5 mm). In this regard, Freedman et al. [59] found that the agreement between BF% measured by DXA or Slaughter equations varied substantially depending on whether they compared the methods in obese or non-obese children. In their study the authors showed that Slaughter equations were accurate among non-obese children, whereas they overestimated BF% in obese children [59]. Technical aspects could constitute another reason why BF% measured by skinfolds differ from BF% assessed by DXA. Moreover, commonly used calipers could involve structural limitations when used in subjects with excess of adiposity. They usually allow maximum diameters of up to 60 mm and cannot report true skinfold thicknesses in subjects with a high excess of adiposity. In addition, differences in our study could also be explained by the different ethnic group, as the sample used to develop the Slaughter equations did not include Latin American youths [19].

This study shows that BF% determined by Seca^®^ mBCA 514, Tanita^®^ BC 420MA, and Slaughter equations is significantly and strongly correlated with measurements assessed by DXA. These data agree with previous studies in which significant correlations were identified between BF% measured by BIA [16,24,60] or Slaughter skinfold-thickness equations [59,61] and DXA in youth with an excess of adiposity. Nevertheless, the fact that the different BF% measurement methods correlate significantly does not imply an agreement between them or that they could replace DXA when assessing BF% [46].

Limitations to the present study should be noted. Firstly, the results obtained from the comparison between methods may not be applicable to other BIA devices. Since validation is a continuous process, additional studies are required for Seca^®^ mBCA 514, Tanita^®^ BC 420MA, Slaughter equations and DXA methods. Secondly, since our cohort comprised a well-characterized sample of Colombian children and adolescents, the results presented here may not be generalizable to other ethnicities. Another potential limitation is that we did not include the phase of the menstrual cycle in the timing of BIA measurements. However, previous studies investigating the effect of the menstrual cycle on body composition determined by BIA devices support that BIA can be used at any time during a woman’s menstrual cycle without altering the body composition values [62,63]. In contrast, the strengths of this study include the use of DXA, a robust and well-accepted means as the standard method and the use of Bland–Altman plots to interpret the results. Additionally, highly standardized procedures have been developed within the HEPAFIT study to avoid measurement bias. Finally, to the best of our knowledge, this is the first study to investigate the agreement between BIA analysers and Slaughter skinfold-thickness equations in comparison with DXA techniques in a population of Latin American children and adolescents with overweight/obesity or excess of adiposity.

## 5. Conclusions

In summary, the present study shows that, compared to DXA, BIA analysers and Slaughter skinfold-thickness equations do not provide accurate measurements of BF% and, therefore, they are not interchangeable methods for evaluating BF% in Latin American children and adolescents with excess of adiposity. BF% measurements assessed by BIA analysers and Slaughter skinfold-thickness equations should be interpreted with caution since they might underestimate BF% in children with obesity. Further studies are needed in order to investigate other valid, reliable, affordable, easily accessible, and safe methods or equations that could replace DXA in the evaluation of BF% in children and adolescents with excess of adiposity.

## Figures and Tables

**Table 1:** Descriptive characteristics of HEPAFIT study participants by sex.

Characteristics	Boys	Girls	*p*-Value
	(*n*= 42)	(*n* = 85) ^a^	
Chronological age (years)	12.9 (1.2)	13.7 (1.7)	0.003
Age of PHV (years)	12.3 (0.6)	14.4 (0.6)	0.001
Weight (kg)	57.3 (9.4)	57.7 (10.4)	0.829
Height (cm)	154.8 (8.4)	155.9 (7.5)	0.486
Sitting height (cm)	77.2 (4.5)	81.1 (4.2)	<0.001
Body mass index (kg/m^2^)	24.2 (2.5)	23.5 (4.1)	0.359
Overweight+obese prevalence (%) *	41.1	55.9	0.066
BF > 30% by DXA prevalence (%) *	97.6	100	0.997
*z*-BMI	1.73 (0.64)	1.39 (0.85)	0.013
Pubertal stage, (genital maturity I to V) (%) *	0.0/43.9/24.4/26.8/4.9	0.0/3.5/25.9/48.2/22.4	0.001
Pubertal stage (pubic hair I to V) (%) *	2.4/46.3/29.3/19.5/2.4	8.2/20.0/27.1/31.8/12.9	0.001
Waist circumference (cm)	79.4 (6.8)	74.6 (8.4)	0.009
**Body fat percentage**			
BF% by DXA	38.0 (4.6)	40.8 (4.1)	0.001
BF% by Seca^®^ mBCA 514	23.4 (6.2)	32.3 (5.8)	<0.001
BF% by Tanita^®^ BC 420MA	24.0 (6.4)	29.5 (6.0)	<0.001
BF% by skinfold-thickness equations	29.0 (7.3)	29.8 (5.8)	0.510

Data are reported as mean values (standard deviation, SD) or percentages. Significant between-sex differences (*t*-tests or * chi-squared test X^2^). Abbreviations: BF%: body fat percentage; z-BMI: *z*-score of body mass index; DXA: dual energy X-ray absorptiometry; PHV: peak height velocity). **^a^** Body fat percentage in DXA, Seca^®^ mBCA 514, and Tanita^®^ BC 420MA measurements were not available for four children (*n* = 81) because some families did not attend the tests. Slaughter et al. (1988) equation [19]: All female: BF% = 1.33 (tric+subsc) − 0.013 (tric+subsc)^2^ − 2.5. Prepubertal male: BF% = 1.21 (tric+subsc) − 0.008 (tric+subsc)^2^ − 1.7. Pubertal male: BF% = 1.21 (tric+subsc) − 0.008 (tric+subsc)^2^ − 3.4 Post-pubertal male: BF% = 1.21 (tric+subsc) − 0.008 (tric+subsc)^2^ − 5.5. All female when (tric+subsc) > 35 mm: BF% = 0.546 (tric+subsc) + 9.7. All male when (tric+subsc) > 35 mm: BF% = 0.783 (tric+subsc) + 1.7. Female: BF% = 0.61 (tric+calf) + 5.1. Male BF% = 0.735 (tric+calf) + 1.

**Table 2:** Mean BF% difference and Lin’s concordance correlation coefficient between DXA and Seca^®^ mBCA 514, Tanita^®^ BC 420MA, and Slaughter skinfold-thickness equations by sex.

**Boys (*n* = 42)**					
**DXA vs. Seca^®^ mBCA 514**		**DXA vs. Tanita^®^ BC 420MA**		**DXA vs. Slaughter equations**	
BF% (95% CI)	*ρ*c (95% CI)	BF% (95% CI)	*ρ*c (95% CI)	BF% (95% CI)	*ρ*c (95% CI)
14.6 *	0.149	14.0 *	0.096	9.0 *	0.227
(13.3–15.9)	(0.078–0.218)	(12.1–15.9)	(0.023–0.168)	(12.1–11.0)	(0.092–0.353)
**Girls (*n* = 85) ^a^**					
**DXA vs. Seca^®^ mBCA 514**		**DXA vs. Tanita^®^ BC 420MA**		**DXA vs. Slaughter equations**	
BF% (95% CI)	*ρ*c (95% CI)	BF% (95% CI)	*ρ*c (95% CI)	BF% (95% CI)	*ρ*c (95% CI)
8.5 *	0.323	11.3 *	0.175	11.1 *	0.179
(7.8–9.3)	(0.241–0.400)	(10.3–12.3)	(0.112–0.237)	(10.3–11.9)	(0.119–0.238)

Differences between BF% values in function of the measurement method (DXA/Seca^®^ mBCA 514, DXA/Tanita^®^ BC 420MA, and DXA/Slaughter equations) were examined using paired sample *t*-tests. * Significant between-methods differences (*p* < 0.001). Abbreviations: BF%: body fat percentage; CI: confidence interval; DXA: dual energy X-ray absorptiometry; *ρc*: Lin’s concordance correlation coefficient. **^a^** Body fat percentage in DXA, Seca^®^ mBCA 514, and Tanita^®^ BC 420MA measurements were not available for four children (*n* = 81) because some families did not attend the tests.
